# Epidemiological Investigation of Animal Brucellosis in Domestic Ruminants in Greece from 2015 to 2022 and Genetic Characterization of Prevalent Strains

**DOI:** 10.3390/pathogens13090720

**Published:** 2024-08-26

**Authors:** Mary Emmanouil, Dimitrios Vourvidis, Anna Kyrma, Sofia Makka, Elina Horefti, Emmanouil Angelakis

**Affiliations:** 1 Diagnostic Department and Public Health Laboratories, Hellenic Pasteur Institute, 11521 Athens, Greece; emmanouilm@pasteur.gr (M.E.); s.makka@pasteur.gr (S.M.); horefti@pasteur.gr (E.H.); 2 Ministry of Rural Development and Food, 15341 Attica, Greece; dvourvidis@gmail.com (D.V.); akyrma@minagric.gr (A.K.)

**Keywords:** Brucellosis, *Brucella* spp., Greece, ruminants, *B. abortus*

## Abstract

Brucellosis is one of the most important zoonotic diseases in Greece, causing a significant burden on both human and animal vitality as well as economic loss. The present study was conducted from 2015 to 2022 on 711,415 serum samples by determining the seroepidemiology of Brucellosis among livestock in 24 geographical areas in Greece using the Rose Bengal Test (RBT) and the complement fixation test (CFT) and further performing genetic analysis of *Brucella* spp. by species-specific real-time PCR and MLVA *Brucella* analysis. A total of 3086 serum samples from goats, sheep, and cattle showed positive results using the RBT and CFT, and only strongly positive samples (n = 800) were preserved in the Βlood Bank of the Veterinary Laboratory of Brucellosis. From these, 212 sera samples were randomly selected for molecular and genetic analysis. The results indicated that the incidence rate of Brucellosis is higher in cattle herds in comparison with other animal species. Overall, 48 samples tested positive by real-time PCR, of which forty-seven of them were *B. abortus* and one was *B. melitensis*. Genetic analysis of two *B. abortus* samples revealed a common pattern, indicating two Bruce04, two Bruce18, four Bruce07, two Bruce09, three Bruce16, and four Bruce30 for both samples, which, interestingly, were not identical with the known genotypes in the public MLVA *Brucella* database. Our findings substantiate that animal Brucellosis remains a health issue in Greece, with a stable but apparent incidence rate, and further investigation is needed to fully characterize the newly identified *Brucella* strains in Greece.

## 1. Introduction

Brucellosis is responsible for one of the most serious and debilitating zoonotic diseases, which globally targets a broad range of mammals, subsuming humans [1]. While the physiological reservoirs of *Brucella* are in the majority of domestic ruminants, the bacterium has managed to cross host species barriers, posing a threat to human health [2]. At present, there are 13 phylogenetically distinguished species of *Brucella*. *B. abortus*, *B. melitensis*, and *B. suis*, which are the predominant species circulating in livestock and the main cause of most human Brucellosis cases [2,3,4].

Despite the eradication of bacterial agents from most parts of the European continent, the infection seems to be persistent in southern Europe, highlighting the countries with intensive livestock farming [5]. In Greece, the average annual frequency of human Brucellosis occurrence is 1.3 incidents per 100,000 population, which is one of the highest indexes in Europe, with most cases reported in rural areas where people have close contact with infected animals, especially among farmers, livestock breeders, and veterinarians [6,7]. It is proven that a human vaccine has great health risks; therefore, continuous surveillance for the bacterium spread in livestock and the establishment of a vaccination program became necessary [8]. Additionally, Brucellosis in animals causes stupendous financial losses due to abortions, premature births, decreases in milk production, and lower reproduction rates [1]. As a result, the country underwent a perceptible partition into two zones, namely, the eradication and the vaccination zone [8].

Accessing the diagnosis field, the “golden standards” are still bacterial isolation and serological tests. Isolation of *Brucella* provides certain limitations due to a long cultivation process, health risks, and skilled personnel demand. Furthermore, negative bacterial isolation does not guarantee that the animal from which the sample was taken is not infected. Serological tests are regularly utilized as diagnostic tools. The most commonly used ones are the Rose Bengal Test (RBT), the complement fixation test (CFT), and the enzyme-linked immunosorbent assay (ELISA) [2]. Every screening test has its own specificity and sensitivity limitations, although when used pairwise, efficacy may increase [1]. In Greece, serological analyses in livestock animals provide efficient annual epidemiological data, indicating a stable yet non-negligible spreading rate.

The classical serological tools and conventional analysis techniques for *Brucella* are not always compensatory for genetic tracing in animals. Molecular analysis is a significant breakthrough; yet, it is not well established [9]. Conventional PCR assays can be designed as a uniplex PCR (detecting a single target) or a multiplex PCR (detecting several targets) in a single test and are commonly used for identifying *Brucella* [10]. However, they are limited in their efficiency and are only capable of identifying the affiliation to the genus or, in the best-case scenario, to one of the species of *Brucella*. These methods are unable to discriminate the main *Brucella* species with accuracy [11]. In contrast, real-time PCR offers several benefits when compared to traditional methods, including its ability to provide rapid results, detect low levels of *Brucella* DNA, differentiate between *Brucella* species (*Brucella* spp.) and other related bacteria, and identify *Brucella* DNA in a variety of clinical samples such as blood, milk and tissue [12]. Several real-time PCR assays, specific to the genus or species, have been developed using primers derived from various gene sequences found in the *Brucella* genome [11,13].

MLVA (Multiple Loci Variable–number tandem repeat Analysis) is the most favorable method for genotyping *Brucella*. It uses PCR amplification to target specific regions of the *Brucella* genome containing variable numbers of tandem repeats. The number of repeats at each locus varies between different *Brucella* strains, which allows differentiation and typing of different isolates. By comparing the MLVA numerical profiles, researchers can determine relatedness and track the spread of the bacteria. Additionally, MLVA data can be used to study the genetic diversity and evolution of *Brucella* populations [14]. The genetic heterogeneity that was revealed by MLVA opposes the commonly accepted notion of genetic uniformity within the genus [15].

The objective of this study is the epidemiological analysis of animal Brucellosis in Greece within the last decade. At the same time, we aim to assess circulating *Brucella* strain genetic profile and characterization using species-specific real-time PCR and an alternative approach of an MLVA *Brucella* analysis. Other epidemiological studies conducted in Greece prior to 2015 have shown the circulation of Brucellosis in livestock and have emphasized the correlations between human Brucellosis and animal disease [16]. The present research introduces new data from 2015 to 2022 in the existing literature, pinpointing that Brucellosis remains a difficult disease to control.

## 2. Materials and Methods

### 2.1. Sample Collection

All samples were acquired from the Veterinary Laboratory of Brucellosis and Bacterial Disease Serological Unit, Department of Diagnostic Pathology, Anatomy, Histology and Microbiology, Directorate of Athens Veterinary Center, Ministry of Rural Development and Food and were kept at −20 °C until the analysis. A total of 711,415 serum samples of goats, sheep, and cattle (Tables S1 and S2) were received from 24 geographical areas in Greece, including both mainland and islands (out of a total of 54 geographical areas) during the period 2015–2022, and serological analysis revealed that 3068 samples were found to be positive to varying degrees of serum and antigen agglutination by the preliminary Rose Bengal Test (RBT: flock/herd screening test). The positive samples were retested by the confirmatory complement fixation test (CFT) and then preserved in frozen conditions at −20 °C until their molecular analysis. The RBT is mainly used to determine the level of livestock healthiness in terms of Brucellosis at the farm level. For the purpose of this study, a sample size of 212 RBT-positive sera of ruminants was randomly selected from 800 RBT strongly positive blood samples preserved in the Βlood Bank of the Veterinary Laboratory of Brucellosis to support diagnostic needs under the framework of the Brucellosis control and eradication national programs. None of the tested animals included in this study had been vaccinated against Brucellosis, and there was no history of abortions at the farm level.

### 2.2. Serological Testing

Animal fresh sera (bovine, sheep, and goats) were tested neat by the Rose Bengal Test (RBT) in the laboratory of Brucellosis (30 μL bovine and 75 μL sheep and goats). Blood samples were centrifuged (1800× *g* for 5 min), and the strongly positive sera were stored in Eppendorf tubes at −20 °C until needed. Serum sample preparation for analysis and the guidelines for performing the diagnostic techniques complied with the requirements of the OIE Manual [17]. For the RBT, which is a rapid agglutination test, a commercially available antigen for veterinary practice is used, and the antigen is stored according to the supplier’s instructions and should never be stored at a temperature ≤ 10 °C. The same volume of neat serum and antigen was placed side by side on a plate and mixed thoroughly and rapidly into a circle, and the reagent was spread over the entire circle on the plate. The plate containing each specimen was rotated by a rocking shaker for 4 min. The presence of agglutination was observed immediately with the naked eye under good lightening. A two-stage confirmatory CFT method was used for retesting the 212 RBT-positive serum samples. In the first, the inactivated test serum is mixed with the antigen and complement. If the animal’s blood serum contains antibodies against *Brucella* spp. bacteria, a complex is formed with the test antigen, which binds to the complement.

### 2.3. Epidemiological Data Analysis

During the period 2015–2022, a total number of 711,415 animals were tested by serological analysis from different regions in Greece. The epidemiological data acquired were subjected to disseverance procedures and subsequently categorized based on the geographical location, the year of acquisition (Tables S1 and S2), the number of animals that were tested, and the number of double-positive test outcomes (RBT and CFT) observed. The occurrence rate of animal Brucellosis in various municipalities of Greece was computed through Microsoft Excel 2021 software (Microsoft, Redmond, WA, USA). Epidemiological maps illustrating the prevalence of this bacterial infection among the Greek livestock population over the past seven years were generated based on the analyzed data.

### 2.4. Molecular Analysis

DNA extraction from the serum samples was performed using the MagCore automated extraction system according to the manufacturer’s instructions. Three sets of real-time PCR amplification were conducted. Firstly, all samples were tested for the presence of *Brucella* spp. in a genus-specific manner [18]. Secondly, all the positive for *Brucella* sp. serums were subtyped as *B. abortus* and *B. melitensis* using primers designed for directly targeting these species [18]. The positive control used in this study is a DNA extract derived from a human whole blood clinical sample cultured in our accredited BSL3 laboratory. Real-time PCR reactions were carried out in 25 μL total volume: 1× real-time PCR Taq mix (PCR Biosystems Ltd., London, UK), forward and reverse primers at a 0.5 μM concentration each, a probe at a 0.25 μM concentration [12], an exogenous internal control [19], and 5 μL of the template and molecular grade H_2_O up to final volume as mentioned.

### 2.5. Genotyping of Brucella Samples

*Brucella*-positive samples by real-time PCR, such as *B. abortus* and *B. melitensis*, were subjected to genotyping using the MLVA-16 approach in an alternating form. Out of the 16 primer panels, the most discriminatory primers were selected [2]. Conventional PCR using each set of primers individually was performed, as per the established protocol [14,15]. The resultant PCR products were purified, quantified, and subjected to Sanger sequencing. Analysis of the data was performed using Chromas Pro software version 11, which enabled the production of an MLVA numerical code for each sample [11].

## 3. Results

### 3.1. Animal Brucellosis Epidemic Profile during 2015–2022

**Figure 1:**
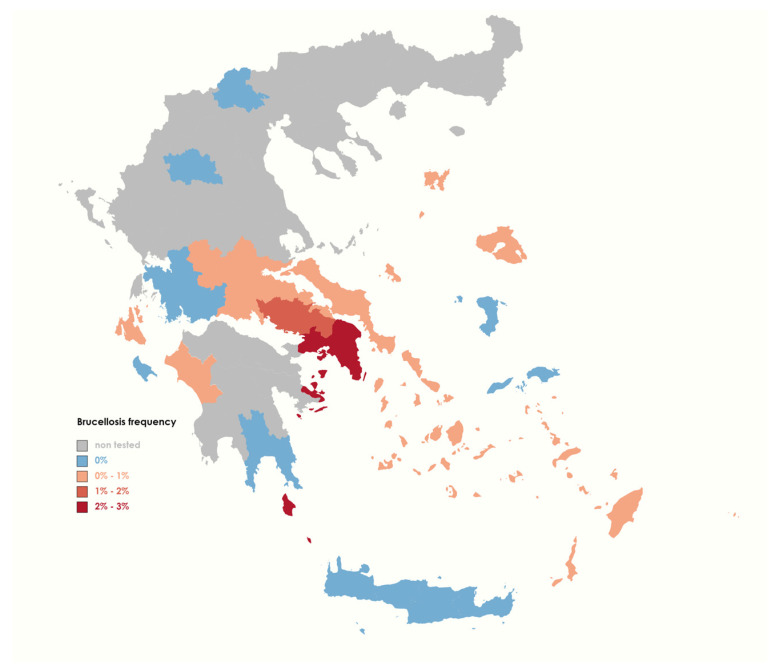
Prevalence of animal Brucellosis among goats and sheep in different regions of Greece during 2015–2022.

**Figure 2:**
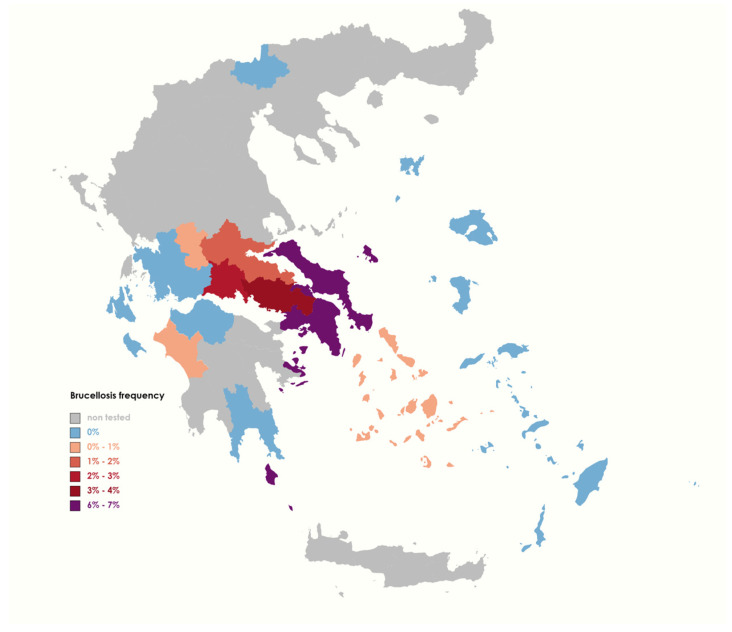
Prevalence of animal Brucellosis among cattle in different regions of Greece during 2015–2022.

From 2015 to 2022, a comprehensive serological examination was conducted in the entire country to detect Brucellosis in livestock animals. During this period, a total number of 711,415 animals were tested. These results were used to generate epidemiological maps. Figure 1 and Figure 2 depict the prevalence of this bacterial infection among the livestock population in various regions of Greece and provide insight into the geographic distribution of animal Brucellosis in the country. Interestingly, the incidence rate of Brucellosis in sheep and goat farms exhibits a consistent annual pattern, whereas this trend is not observed in cattle herds (Tables S1 and S2).

Table 1 describes the serological test results for goats and sheep in each municipality of Greece from 2015 to 2022. The results of serological tests show that the region of Attica has constantly exhibited the highest score (2.25%) in comparison to other regions. Conversely, certain regions such as Chania, Heraklion, Rethymno, and Lasithi in Crete exhibit promising signs of *Brucella* eradication, as none of the 219,217 samples collected in these areas during the specified period tested positive for the pathogen (Table 1). The infection’s persistence in central Greece resulted in a noticeable outbreak in sheep and goat farms during 2019 in Attica (Table S1), which ceased the following year.

Based on the epidemiological data, the incidence rate of Brucellosis was higher in cattle herds compared to the other animal populations studied (Figure 1 and Figure 2). As also shown in Table 2, Attica, Euboea, Boeotia, Phokis, and Pthiotis were among the regions most severely impacted by Brucellosis, with positivity rates of 6.7%, 6.5%, 3.8%, 2.8%, and 1.4%, respectively. In cattle, our data indicated the presence of possible outbreaks in Euboea in 2015 (16.3%) and Boeotia in 2016 (15.7%), while Attica exhibited continuously high infection rates until 2020 (Table S2).

### 3.2. Real-Time PCR Analysis

Real-time PCR analysis to detect the presence of *Brucella* DNA was performed on a total of 212 serum samples that were identified as *Brucella* IgM/IgG positive using the established serological techniques of the RBT and CFT as previously mentioned. Of the total of 212 serum samples used for molecular analysis, 115 samples belong to cattle and 97 samples to small ruminants (sheep and goats). Among the 115 bovine serum samples, in 19, we detected *Brucella* spp. DNA by real-time PCR, and among 97 serum samples from small ruminants, we found 29 positive samples. A total of 48 serum samples were found to be real-time PCR positive (23% of the tested positive samples), albeit at relatively high cycle threshold (Ct) values, which suggests a relatively low quantity of *Brucella* sp. DNA present. The main reasons for relatively low PCR positivity are the stage of the disease that may lead to low bacteremia load and the type of samples tested. The next step included subtyping the most predominant *Brucella* species, *abortus* and *melitensis*. Out of the 48 serum specimens, forty-seven were characterized as *B. abortus* using the species-specific primers for accurate identification and one as *B. melitensis*. The only positive sample for *B. melitensis* originated from a sheep/goat herd.

### 3.3. MLVA Analysis

The two *B. abortus* samples with the lowest (Ct) values were selected for genotyping. Sanger sequencing was performed on the samples after the conventional PCRs, and the resulting sequences were identified. Based on the number of observed repeats, the numerical codes for the *Brucella* MLVA could be determined as two Bruce0, two Bruce18, four Bruce07, two Bruce09, three Bruce16, and four Bruce30 for both serum samples (Table 3) [1,11]. The numerical profiles of the investigated strains were found to be non-identical with the known genotypes present among the 7212 strains available at the moment in the public MLVA *Brucella* database accessed on 15 January 2024 (https://microbesgenotyping.i2bc.paris-saclay.fr/databases/view/61). Further investigation is needed to fully characterize the newly identified *Brucella* strains in Greece. Previous studies using in silico genotyping also revealed novel subgenotypes of the East Mediterranean lineages comprising Greek strains, indicating the importance of further genetic analysis of the newly identified strains [20].

## 4. Discussion

Brucellosis is a zoonotic disease caused by the bacteria of *Brucella* genus, and it is transmitted to humans through direct contact with ill animals or consumption of infected dairy products [21]. In Greece, Brucellosis is still considered a pressing public health concern, with an abundant annual incident not only causing a significant burden on both human and animal vitality but also affecting the national economy [22,23]. In this study, we aimed to investigate the epidemiological profile of animal Brucellosis, adding to the frequency and distribution of *Brucella* sp. in livestock farming, and to further inspect its genetic characteristics in Greece.

Our epidemiological findings substantiate that animal Brucellosis remains a difficult to control and eradicate consequential health issue in Greece, with a stable but apparent incidence rate [16]. This indicates a persistent burden of the infection in animals and, thereafter, humans over time. Interestingly, a noticeable disparity in the incidence rates of Brucellosis was noticed between goats/sheep and cattle, with the latter displaying considerably higher disease rates (Table 1 and Table 2), especially in the Attica region. This fact may demonstrate potential variances in the prevalence as well as the transmission dynamics of this pathogen among livestock populations. It is acknowledged that *B. abortus* is predominantly linked to infections in cattle [24], while *B. melitensis* is primarily associated with infections in small ruminants, such as goats and sheep [24,25]. Consequently, the infectious dynamic of *B. abortus* may be higher than that of *B. melitensis*. Our results are consistent with expectations, as *B. abortus* and *B. melitensis* are the predominant species circulating in livestock in Greece. The molecular characterization of *Brucella* spp. can be performed in whole blood or serum samples, although serum samples are not the optimal approach. In this study, molecular analysis of serum samples from seropositive cattle and small ruminants was performed retrospectively; thus, blood samples were not available. In 23% of the seropositive animals tested, *Brucella* spp. DNA was detected in the serum sample by molecular methods.

Moreover, the frequency of Brucellosis in goat and sheep populations has been observed to be comparatively diminutive, with incidence rates remaining below 1% (for the whole country) over this seven-year period. However, in spite of the relatively low occurrence rates, the persistence of the infection in several geographic areas throughout the Greek domain, particularly in central Greece (Figure 1), suggests a scarcity of effective eradication or control strategies. Conversely, there are indications that certain districts of Greece may be free of Brucellosis in their sheep and goat populations. Notably, the island of Crete (Chania, Heraklion, Lasithi, Rethymno) has exhibited a total absence of positive Brucellosis serological findings during our study initiatives (Figure 1). An interesting observation is that regions where immediate slaughter of the whole livestock, infected goat and sheep or not [8], is implemented, rather than relying solely on vaccination, have exhibited a complete elimination of Brucellosis bacteria. This suggests that a combination of additional control measures, including proper removal of infected animals or enhancement of the vaccine’s efficacy, may be essential to achieve the annihilation of the disease in the rest of the country.

In the cattle field, the spatial analysis of the data collected has provided a contrasting perspective on the epidemiology of Brucellosis disease in the country (Figure 2). Specifically, the rate of serologically diagnosed Brucellosis in cattle was found to be significantly higher (3.4%, Table 2) compared to the goat–sheep Brucellosis incidents. This finding poses serious ramifications for the prevention and control of Greek programs, as it provides insight that disease may be more extensive and endemic than formerly known. Another paradigm of under-diagnosis in domestic ruminant populations in Greece is the case of widespread exposure to *C. burnetii*. In a previous study, we have provided evidence of *C. burnetii* seropositivity in productive animals throughout most Greek territories [26].

The period 2020–2021, marked by the emergence of the SARS-CoV-2 virus, witnessed a reduction in the research output across the whole scientific landscape. Despite this global tendency, the collected data of the present study indicate that the pandemic era did not have a significant influence, as the number of animals tested stayed consistent with that of preceding years. This observation suggests that the research results remain valid and representative of the animal population in the study area.

## 5. Conclusions

In conclusion, this study on *Brucella* epidemiology has shed light on several important factors regarding the incidence and distribution of this infectious bacteria in Greek livestock. The findings suggest that *Brucella* infections continue to pose a significant animal and subsequently public health threat in many parts of the country, particularly in rural areas where animal husbandry is a common occupation. This study highlights the need for improved surveillance and control measures to effectively manage and prevent the spread of Brucellosis. Ultimately, this study provides valuable insights into the epidemiology of *Brucella* infections in animals that can inform policy and public health interventions aimed at reducing the burden and the spread of the disease.

## Figures and Tables

**Table 1:** Goats and sheep serological test results expressed as Brucellosis positivity in each municipality of Greece tested from 2015 to 2022.

Region	Tested	Positive	Positivity (%)
Aetolia–Acarnania	195	0	0
Attica	24,771	557	2.25
Boeotia	5317	63	1.18
Cephalonia	115,234	15	0.01
Chania	25,388	0	0
Chios	8588	0	0
Cyclades	133,591	2	0.001
Dodekanese	42,633	128	0.3
Elis	129	1	0.78
Euboea	22,787	98	0.43
Evrytania	5910	1	0.02
Grevena	49	0	0
Heraklion	34,637	0	0
Laconia	37	0	0
Lasithi	8240	0	0
Lesbos	26,618	32	0.12
Pella	60	0	0
Phocis	796	1	0.13
Pthiotis	33,813	202	0.01
Rethymno	150,952	0	0
Samos and Ikaria	12,055	0	0
Zakynthos	2399	0	0
Total	654,199	1100	0.17

**Table 2:** Cattle serological test results expressed as Brucellosis positivity in each municipality of Greece tested from 2015 to 2022.

Region	Tested	Positive	Positivity (%)
Achaea	116	0	0
Aetolia–Acarnania	297	0	0
Attica	12,914	900	6.97
Boeotia	22,487	861	3.8
Cephalonia	311	0	0
Chios	177	0	0
Cyclades	10,552	3	0.03
Dodekanese	2329	0	0
Euboea	1972	129	6.54
Evrytania	750	1	0.13
Kilkis	35	0	0
Laconia	13	0	0
Lesbos	117	0	0
Phokis	760	21	2.76
Pthiotis	3938	53	1.35
Samos and Ikaria	443	0	0
Zakynthos	5	0	0
Total	57,216	1968	3.4

**Table 3:** MLVA data results. The exact primer sequences used and the repeated sequences, as well as the number of repeats (NORs) found, are depicted.

Primer Set	Forward Seq 5′-3′	Reverse Seq 5′-3′	Gene Locus	Repeat	NOR Sample A	NOR Sample B
Bruce 04	CTGACGAAGGGAAGGCAATAAG	CGATCTGGAGATTATCGGGAAG	BMEI1496	AGGGCAGT	2	2
Bruce 07	GCTGACGGGGAAGAACATCTAT	ACCCTTTTTCAGTCAAGGCAAA	BMEI1204	GAATAGGG	4	4
Bruce 09	GCGGATTCGTTCTTCAGTTATC	GGGAGTATGTTTTGGTTGTACATAG	BMEI0570	AGGGCAGT	2	2
Bruce 16	ACGGGAGTTTTTGTTGCTCAAT	GGCCATGTTTCCGTTGATTTAT	BMEII0523	TAAGGGAG	3	3
Bruce 18	TATGTTAGGGCAATAGGGCAGT	GATGGTTGAGAGCATTGTGAAG	BMEII0326	AGTAAGGG	2	2
Bruce 30	TGACCGCAAAACCATATCCTTC	TATGTGCAGAGCTTCATGTTCG	downstream of BMEI1453	AGGGCAGT	4	4

## Data Availability

The original contributions presented in the study are included in the article/Supplementary Materials, and further inquiries can be directed to the corresponding author.

## Supplementary Materials

The following supporting information can be downloaded at https://www.mdpi.com/article/10.3390/pathogens13090720/s1, Table S1: Annual results for goats and sheep serological tests of Brucellosis in each municipality of Greece tested from 2015 to 2022; Table S2: Annual results for cattle serological tests of Brucellosis in each municipality of Greece tested from 2015 to 2022.

## References

[1] Gwida M., Al Dahouk S., Melzer F., Rösler U., Neubauer H., Tomaso H. (2010). Brucellosis—Regionally Emerging Zoonotic Disease?. Croat. Med. J..

[2] Wareth G., El-Diasty M., Melzer F., Schmoock G., Moustafa S.A., El-Beskawy M., Khater D.F., Hamdy M.E.R., Zaki H.M., Ferreira A.C. (2020). MLVA-16 Genotyping of Brucella abortus and Brucella melitensis isolates from Different Animal Species in Egypt: Geographical Relatedness and the Mediterranean Lineage. Pathogens.

[3] Baldwin C.L., Goenka R. (2006). Host Immune Responses to the Intracellular Bacteria Brucella: Does the Bacteria Instruct the Host to Facilitate Chronic Infection?. Crit. Rev. Immunol..

[4] Jamil T., Akar K., Erdenlig S., Murugaiyan J., Sandalakis V., Boukouvala E., Psaroulaki A., Melzer F., Neubauer H., Wareth G. (2022). Spatio-Temporal Distribution of Brucellosis in European Terrestrial and Marine Wildlife Species and Its Regional Implications. Microorganisms.

[5] Garofolo G., Di Giannatale E., De Massis F., Zilli K., Ancora M., Cammà C., Calistri P., Foster J.T. (2013). Investigating genetic diversity of Brucella abortus and Brucella melitensis in Italy with MLVA-16. Infect. Genet. Evol..

[6] Lytras T., Danis K., Dounias G. (2016). Incidence Patterns and Occupational Risk Factors of Human Brucellosis in Greece, 2004–2015. Int. J. Occup. Environ. Med..

[7] European Centre for Disease Prevention and Control (2023). Brucellosis. ECDC. Annual Epidemiological Report for 2021.

[8] Katsiolis A., Papanikolaou E., Stournara A., Giakkoupi P., Papadogiannakis E., Zdragas A., Giadinis N.D., Petridou E. (2022). Molecular detection of Brucella spp. in ruminant herds in Greece. Trop. Anim. Health Prod..

[9] Khan M.Z., Zahoor M. (2018). An Overview of Brucellosis in Cattle and Humans, and its Serological and Molecular Diagnosis in Control Strategies. Trop. Med. Infect. Dis..

[10] Kumar V., Bansal N., Nanda T., Kumar A., Kumari R., Maan S. (2019). PCR Based Molecular Diagnostic Assays for Brucellosis: A Review. Int. J. Curr. Microbiol. Appl. Sci..

[11] Kurmanov B., Zincke D., Su W., Hadfield T.L., Aikimbayev A., Karibayev T., Berdikulov M., Orynbayev M., Nikolich M.P., Blackburn J.K. (2022). Assays for Identification and Differentiation of Brucella Species: A Review. Microorganisms.

[12] Hinić V., Brodard I., Thomann A., Cvetnić Ž., Makaya P.V., Frey J., Abril C. (2008). Novel identification and differentiation of *Brucella melitensis*, *B. abortus*, *B. suis*, *B. ovis*, *B. canis*, and *B. neotomae* suitable for both conventional and real-time PCR systems. J. Microbiol. Methods.

[13] Hinić V., Brodard I., Thomann A., Holub M., Miserez R., Abril C. (2009). IS711-based real-time PCR assay as a tool for detection of *Brucella* spp. in wild boars and comparison with bacterial isolation and serology. BMC Vet. Res..

[14] Le Flèche P., Jacques I., Grayon M., Al Dahouk S., Bouchon P., Denoeud F., Nöckler K., Neubauer H., Guilloteau L.A., Vergnaud G. (2006). Evaluation and selection of tandem repeat loci for a Brucella MLVA typing assay. BMC Microbiol..

[15] Al Dahouk S., Le Flèche P., Nöckler K., Jacques I., Grayon M., Scholz H.C., Tomaso H., Vergnaud G., Neubauer H. (2007). Evaluation of Brucella MLVA typing for human brucellosis. J. Microbiol. Methods.

[16] Fouskis I., Sandalakis V., Christidou A., Tsatsaris A., Tzanakis N., Tselentis Y., Psaroulaki A. (2018). The epidemiology of Brucellosis in Greece, 2007–2012: A ‘One Health’ approach. Trans. R. Soc. Trop. Med. Hyg..

[17] World Organization of Animal Health (2022). Terrestrial Manual 2018, Chapter 3.1.4.—Brucellosis (Infection with Brucella abortus, B. melitensis and B. suis).

[18] Probert W.S., Schrader K.N., Khuong N.Y., Bystrom S.L., Graves M.H. (2004). Real-time multiplex PCR assay for detection of *Brucella* spp., *B. abortus*, and *B. melitensis*. J. Clin. Microbiol..

[19] Nolan T., Hands R.E., Ogunkolade W., Bustin S.A. (2006). SPUD: A quantitative PCR assay for the detection of inhibitors in nucleic acid preparations. Anal. Biochem..

[20] Brangsch H., Sandalakis V., Babetsa M., Boukouvala E., Ntoula A., Makridaki E., Christidou A., Psaroulaki A., Akar K., Gürbilek S.E. (2023). Genotype diversity of brucellosis agents isolated from humans and animals in Greece based on whole-genome sequencing. BMC Infect. Dis..

[21] Pal M., Gizaw F., Fekadu G., Alemayehu G., Kandi V. (2017). Public Health and Economic Importance of Bovine Brucellosis: An Overview. Am. J. Epidemiol. Infect. Dis..

[22] Katsiolis A., Papadopoulos D.K., Giantsis I.A., Papageorgiou K., Zdragas A., Giadinis N.D., Petridou E. (2022). *Brucella* spp. distribution, hosting ruminants from Greece, applying various molecular identification techniques. BMC Vet. Res..

[23] Pappas G., Papadimitriou P., Akritidis N., Christou L., Tsianos E.V. (2006). The new global map of human brucellosis. Lancet Infect. Dis..

[24] Godfroid J. (2017). Brucellosis in livestock and wildlife: Zoonotic diseases without pandemic potential in need of innovative one health approaches. Arch. Public Health.

[25] Seleem M.N., Boyle S.M., Sriranganathan N. (2010). Brucellosis: A re-emerging zoonosis. Vet. Microbiol..

[26] Vourvidis D., Kyrma A., Linou M., Edouard S., Angelakis E. (2021). Sero-epidemiology investigation of Coxiella burnetii in domestic ruminants throughout most Greek regions. Vet. Med. Sci..

