# Supplementary material for: Epidemiological Investigation of Animal Brucellosis in Domestic Ruminants in Greece from 2015 to 2022 and Genetic Characterization of Prevalent Strains
Source: Pathogens. 2024 Aug 26;13(9):720. doi: 10.3390/pathogens13090720 (PMC11434918; doi:10.3390/pathogens13090720)
Supplement: Supplementary file 1 [file pathogens-13-00720-s001.zip › pathogens-3140073-supplementary.pdf]

Supplementary Materials:

Table S1. Annual results for Goats and sheep serological tests of brucellosis in each municipality of Greece tested from 2015 to 2022.

REGION	2015			2016			2017			2018			2019			2020			2021			2022		
	TESTED	POSITIVE	POSITIVITY (%)	TESTED	POSITIVE	POSITIVITY (%)	TESTED	POSITIVE	POSITIVITY (%)	TESTED	POSITIVE	POSITIVITY (%)	TESTED	POSITIVE	POSITIVITY (%)	TESTED	POSITIVE	POSITIVITY (%)	TESTED	POSITIVE	POSITIVITY (%)	TESTED	POSITIVE	POSITIVITY (%)
AETOLIA-ACARNANIA	161	0	0	24	0	0	6	0	0	2	0	0	0	0	0	2	0	0	0	0	0	0	0	0
ATTICA	2682	19	0,71	2957	11	0,37	2899	12	0,41	2974	28	0,94	3796	462	12,17	4215	6	0,14	3718	16	0,59	1530	3	0,2
BOEOTIA	125	0	0	309	9	2,91	621	18	0	1192	19	1,59	174	0	0	2445	15	0,61	327	0	0	124	2	1,61
CEPHALONIA	14573	0	0	16577	0	0	29296	15	0	14678	0	0	10326	0	0	12859	0	0	13445	0	0	3480	0	0
CHANIA	35	0	0	0	0	0	7	0	0	42	0	0	0	0	0	13080	0	0	12224	0	0	0	0	0
CHIOS	4943	0	0	304	0	0	420	0	0	0	0	0	0	0	0	1384	0	0	1537	0	0	0	0	0
CYCLADES	14262	0	0	29029	0	0	24408	1	0	15809	0	0	9529	0	0	11309	0	0	17952	1	0,01	11293	0	0
DODEKANESE	15178	80	0,53	27283	48	0,18	166	0	0	0	0	0	0	0	0	6	0	0	0	0	0	0	0	0
ELIS	129	1	0,78	0	0	0	0	0	0	0	0	0	0	0	0	0	0	0	0	0	0	0	0	0
EUBOEA	3408	35	1,03	3952	10	0,25	2820	2	0	1996	6	0,3	3942	21	0,53	3274	14	0,43	2281	6	0,26	1114	4	0,36
EVROS	0	0	0	0	0	0	0	0	0	0	0	0	0	0	0	1	0	0	0	0	0	0	0	0
EVRYTANIA	1259	0	0	792	1	0,13	1136	0	0	733	0	0	138	0	0	791	0	0	514	0	0	547	0	0
GREVENA	12	0	0	0	0	0	28	0	0	0	0	0	0	0	0	9	0	0	0	0	0	0	0	0
HERAKLION	8	0	0	0	0	0	26	0	0	4819	0	0	9129	0	0	13336	0	0	7319	0	0	0	0	0
LACONIA	35	0	0	0	0	0	2	0	0	0	0	0	0	0	0	0	0	0	0	0	0	0	0	0
LASITHI	0	0	0	0	0	0	0	0	0	0	0	0	1	0	0	5402	0	0	2837	0	0	0	0	0
LESBOS	716	0	0	4416	1	0,02	2147	22	0	3182	0	0	4560	0	0	5164	6	0,12	4748	3	0,06	1685	0	0
PELLA	0	0	0	0	0	0	0	0	0	0	0	0	0	0	0	60	0	0	0	0	0	0	0	0
PHOCIS	72	0	0	41	0	0	59	1	0	515	0	0	35	0	0	23	0	0	43	0	0	8	0	0
PTHIOTIS	4881	19	0,39	4731	35	0,74	4730	62	0	5101	25	0,49	4348	19	0,44	4242	19	0,45	3688	4	0,11	2092	19	0,91
RETHYMNO	0	0	0	0	0	0	0	0	0	36933	0	0	42805	0	0	33452	0	0	37762	0	0	0	0	0
SAMOS & IKARIA	5338	0	0	1410	0	0	938	0	0	1396	0	0	1199	0	0	343	0	0	570	0	0	861	0	0
ZAKYNTHOS	293	0	0	1011	0	0	238	0	0	657	0	0	0	0	0	24	0	0	153	0	0	23	0	0
**TOTAL**	**68110**	**154**	**0,23**	**92836**	**115**	**0,12**	**69947**	**133**	0,19	**90029**	**78**	**0,09**	**89982**	**502**	**0,56**	**111421**	**60**	**0,05**	**109118**	**30**	**0,03**	**22757**	**28**	**0,12**

Table S2. Annual results for cattle serological tests of brucellosis in each municipality of Greece tested from 2015 to 2022.

REGION	2015			2016			2017			2018			2019			2020			2021			2022		
	TESTED	POSITIVE	POSITIVITY (%)	TESTED	POSITIVE	POSITIVITY (%	TESTED	POSITIVE	POSITIVITY (%)	TESTED	POSITIVE	POSITIVITY (%	TESTED	POSITIVE	POSITIVITY (%)	TESTED	POSITIVE	POSITIVITY (%)	TESTED	POSITIVE	POSITIVITY (%)	TESTED	POSITIVE	POSITIVITY (%)
ACHAEA	0	0	0	0	0	0	0	0	0	116	0	0	0	0	0	0	0	0	0	0	0	0	0	0
ATTICA	1640	146	8,9	1387	167	12,04	2079	281	13,99	1337	100	7,48	2017	71	3,52	904	64	7,08	763	0	0	2787	71	2,55
DODEKANESE	273	0	0	634	0	0	655	0	0	767	0	0	0	0	0	0	0	0	0	0	0	0	0	0
AETOLIA-ACARNANIA	30	0	0	86	0	0	42	0	0	26	0	0	43	0	0	5	0	0	25	0	0	40	0	0
EUBOEA	741	121	16,33	212	6	2,83	241	1	0,41	412	0	0	186	1	0,57	53	0	0	18	0	0	109	0	0
EVRYTANIA	5	0	0	65	0	0	96	0	0	127	0	0	108	0	0	74	0	0	116	0	0	159	1	1
PHOKIS	0	0	0	170	0	0	142	21	14,79	188	0	0	0	0	0	0	0	0	23	0	0	237	0	0
PTHIOTIS	281	0	0	468	0	0	116	1	0,86	1110	13	1,17	741	0	0	67	39	58,2	935	0	0	220	0	0
CHIOS	11	0	0	0	0	0	45	0	0	0	0	0	40	0	0	81	0	0	0	0	0	0	0	0
CEPHALONIA	0	0	0	0	0	0	0	0	0	98	0	0	121	0	0	0	0	0	92	0	0	0	0	0
KILKIS	0	0	0	0	0	0	35	0	0	0	0	0	0	0	0	0	0	0	0	0	0	0	0	0
CYCLADES	614	0	0	823	0	0	2188	3	0,14	1452	0	0	915	0	0	1222	0	0	1725	0	0	1613	0	0
LACONIA	13	0	0	0	0	0	0	0	0	0	0	0	0	0	0	0	0	0	0	0	0	0	0	0
LESBOS	0	0	0	0	0	0	0	0	0	0	0	0	0	0	0	13	0	0	104	0	0	0	0	0
SAMOS & IKARIA	15	0	0	2	0	0	151	0	0	153	0	0	112	0	0	10	0	0	0	0	0	0	0	0
BOEOTIA	2996	27	0,9	5151	807	15,67	2368	19	0,8	3539	0	0	2885	0	0	875	8	0,91	2654	0	0	2019	0	0
ZAKYNTHOS	0	0	0	0	0	0	0	0	0	0	0	0	0	0	0	0	0	0	5	0	0	0	0	0
**TOTAL**	**6619**	**294**	**4,44**	**8998**	**980**	**10,89**	**8248**	**354**	**4,29**	**9327**	**117**	**1,25**	**7180**	**78**	**1,09**	**3308**	**111**	**3,35**	**6470**	**0**	**0**	**7184**	**72**	**1**
